# Clinical outcomes of ultrasound-guided mammotome vacuum-assisted excision for benign breast tumors and its impact on serum tumor markers

**DOI:** 10.1186/s12893-025-03293-x

**Published:** 2025-11-21

**Authors:** Yong He, Qiong Sun, Chang Zhou, Rong Liu, Lu-Lu Zou, Hua Chen

**Affiliations:** 1 Department of Ultrasound, Yichang Central People’s Hospital (First Clinical Medical College of Three Gorges University), Yichang, 443000 Hubei China; 2 Department of Geriatrics, Yichang City Mental Health Center, 2 Xiamen Road, High-tech District, Yichang, 443000 Hubei China

**Keywords:** Benign breast lesion, Mammotome, Vacuum-assisted excision, CA 15 − 3, Tumor markers, Minimally invasive surgery

## Abstract

**Background:**

Ultrasound-guided vacuum-assisted excision (VAE) is widely used for benign breast lesions, but its systemic impact is unclear. We tested whether VAE increases serum CA15-3 at 3 months and described clinical outcomes.

**Methods:**

In this prospective single-center cohort of 80 consecutive women with BI-RADS 3/4a lesions ≤ 30 mm undergoing Mammotome^®^ VAE, serum cancer antigen (CA)15 − 3, carcinoembryonic antigen (CEA), and CA125 were measured pre-op, 24 h, 1 month, and 3 months. Outcomes included technical success, 30-day complications, BREAST-Q satisfaction (1/3 months), 1-month lesion-volume change, and 12-month local recurrence. Primary endpoint: paired CA15-3 change at 3 months; noninferiority margin + 2 U/mL.

**Results:**

Technical success was achieved in 79/80 cases (98.8%, 95% CI: 93.3%–99.9%). Mean procedure time was 18 ± 5 min; Clavien-Dindo I–II complications occurred in seven patients (8.8%). Baseline CA 15 − 3 was 12.0 ± 4.0 U/mL and 11.7 ± 3.8 U/mL at 3 months (mean Δ − 0.3 U/mL, 95% CI: − 0.8 to 0.2, *p* = 0.24), satisfying the predefined non-inferiority margin. CEA and CA 125 likewise showed no clinically relevant change; the slight CA 125 decline (–1.0 U/mL, *p* = 0.040) lost significance after Bonferroni correction. Lesion-volume reduction at 1 month averaged 93% ± 6%, and BREAST-Q satisfaction at 3 months was 90 ± 7. One local recurrence (1.4%) was detected during 12-month surveillance.

**Conclusions:**

Mammotome^®^ VAE achieved high technical success, low morbidity, excellent cosmesis, and no increase in serum CA15-3, CEA, or CA125 through 3 months. Twelve-month local control was favorable. Longer follow-up and multicenter studies with broader biologic measures are needed to confirm long-term systemic neutrality and external validity.

**Supplementary Information:**

The online version contains supplementary material available at 10.1186/s12893-025-03293-x.

## Introduction

Benign breast lesions, including fibroadenoma, adenosis, papilloma, and benign phyllodes tumors, are prevalent globally and in China. These lesions, while non-cancerous, can mimic malignant tumors [1, 2]. Management of benign breast lesions ranges from observation to excision Emerging minimally invasive options, such as ultrasound-guided VAE, have shown promise as effective alternatives [3, 4]. Studies have demonstrated that VAE achieves complete-removal rates exceeding 90%, making it a highly effective option for lesions up to 3 cm in size [5, 6]. These procedures are well-tolerated and preferred by patients, as they avoid the invasiveness of traditional surgery while effectively managing the lesions [3]. While these local outcomes are well established, the systemic biological impact of VAE has been little studied.

Circulating tumor‑associated antigens (CA 15 − 3, CEA and CA 125) are detectable at low concentrations in benign breast disease. Peri‑operative fluctuations may reflect tissue trauma or transient inflammation rather than oncologic risk. Removal of these lesions by VAE with minimal parenchymal disruption should remain stable after the procedure, but peri‑operative serum marker trajectories remain under‑characterized.

Although tumor markers are not recommended for routine evaluation of benign breast lesions, they are sometimes available from health‑check panels or pre‑operative laboratories. Our aim is to determine whether ultrasound‑guided VAE perturbs circulating antigens. Demonstrating stable CA 15‑3, CEA, and CA 125 after VAE provides reassurance to interventionalists, surgeons, and laboratory medicine teams that routine post‑VAE marker testing is unnecessary when baseline values are normal and histology is benign. To address the residual knowledge gap, we conducted a prospective cohort with four standardized timepoints (baseline, 24 h, 1 month, 3 months) and a prespecified, directional noninferiority hypothesis that 3‑month CA 15‑3 would not increase by more than + 2 U/mL. We also captured 12‑month local control and patient‑reported outcomes to contextualize systemic biomarker behavior.

## Methods

### Study design and setting

We conducted a prospective, self‑controlled cohort at a single center with uniform imaging, technique, and assays (March–November 2023). The protocol was approved by the Yichang City Mental Health Center ethics committee. Written informed consent was obtained. The primary endpoint was the paired change in CA 15‑3 at 3 months with a prespecified noninferiority margin of + 2 U/mL. Secondary endpoints included CEA/CA 125 time courses, technical success, 30‑day complications (Clavien–Dindo), pain at 24 h, BREAST‑Q satisfaction, and 12‑month local recurrence. Detailed ultrasound acquisition parameters and laboratory procedures are provided in the Supplement.

### Participants

Consecutive women aged 18–60 years with one or more BI-RADS 3–4 A breast lesions ≤ 30 mm who were scheduled for ultrasound-guided VAE under local anesthesia were screened. Inclusion required normal baseline serum CA 15 − 3, CEA and CA 125, histological confirmation of benignity by either pre-operative core biopsy or post-excision pathology, and written informed consent. Exclusion criteria comprised a history of malignancy, pregnancy or lactation, autoimmune or hepatic disorders known to affect tumor-marker levels, coagulopathy, and inability to comply with visits. All reasons for exclusion were documented to ensure transparency and enable assessment of potential selection bias.

To limit selection bias, we enrolled consecutive eligible patients, prospectively logged all exclusion reasons, and prespecified analytic handling of missing data. Overall missing of biomarker timepoints was 4.2%, and multiple imputation (m = 5) yielded identical conclusions to complete‑case analyses.

### Study procedures

At Visit 0 (≤ 7 days pre-op) patients underwent clinical assessment, high-resolution breast ultrasound (Mindray Resona 7) and venous blood sampling for CA 15 − 3, CEA, CA 125, complete blood count and biochemistry. VAE (Visit 1) was performed with an 8-gauge Mammotome^®^ probe under real-time ultrasound guidance after infiltration of 1% lidocaine; lesion dimensions, excised tissue volume (measured by volumetric displacement), procedure time (skin-to-skin) and anesthetic dose were recorded in the electronic case-report form (eCRF). All VAEs were performed by a single attending breast interventional sonologist, with more than 5 years of post‑training experience. Haemostasias was achieved by probe-track compression and a sterile compression dressing was applied for 4 h. Standard oral antibiotics (cefuroxime 0.25 g bid × 3 days) and acetaminophen as-needed analgesia were prescribed. Follow-up visits comprised: Visit 2 (24 h ± 6 h) for repeat serum markers, pain VAS and early adverse-event check; Visit 3 (1 month ± 7 days) for ultrasound of the residual cavity, BREAST-Q cosmetic questionnaire (score 0–100; higher values indicate greater satisfaction) [7, 8] and tumor-markers; Visit 4, the primary end-point (3 months ± 14 days) repeating all assessments; and Visits 5–6 (6 and 12 months) for ultrasound-detected recurrence, late complications and satisfaction.

#### Ultrasound acquisition and measurement standardization

All examinations were performed on a Mindray Resona 7 by one of two attending breast sonologists (≥ 5 years’ experience) using a standardized preset (frequency 10–15 MHz; harmonics on; uniform dynamic range). Lesion size was recorded as the maximum diameter on radial and antiradial planes with outer‑edge‑to‑outer‑edge calipers; when three orthogonal diameters were available, volume was computed with the prolate‑ellipsoid formula (0.523 × L × W × H). Complete excision at the index procedure was defined a priori as no residual echogenic lesion within the cavity on real‑time ultrasound and no internal Doppler flow.

#### Follow‑up interpretation

Follow‑up scans (24 h, 1 month, 3 months, 6/12 months) used the same equipment and presets. Local recurrence was defined by a new solid or complex mass at the index site with BI‑RADS‑consistent features warranting tissue diagnosis. We did not implement independent blinded rereads or a formal inter‑observer variability study; this is acknowledged in Limitations and will be addressed with blinded central adjudication and ICC in future multicenter work.

### Outcome measures

The primary outcome was the paired change in serum CA 15 − 3 from baseline to three months, with a non-inferiority margin of ± 2 U/mL to represent a clinically trivial shift that exceeds expected analytic/short‑term biological variability yet would not alter management when absolute values are well below the laboratory upper‑reference limit. In our cohort, 2 U/mL corresponds to ~ 0.5 SD of the baseline CA 15‑3 distribution (12.0 ± 4.0 U/mL), ensuring that only small, non‑actionable changes could be labeled noninferior. Analytically, the Elecsys CA 15‑3 II assay shows repeatability ≈ 1–3% and intermediate precision ≈ 3–5% across low–moderate concentrations; biologically, within‑subject variability (CVi) for CA 15‑3 in healthy individuals is typically ≈ 4–6%. Combined in the reference‑change value (RCV) framework, this yields RCV ≈ 15% at usual baselines—about 1.8–2.0 U/mL at 12 U/mL—supporting + 2 U/mL as a noise‑exceeding, non‑actionable threshold [9, 10]. The biomarker endpoint was intentionally set at 3 months to evaluate peri‑operative and early sustained systemic effects. Markers were obtained at 24 h, 1 month, and 3 months to detect any transient perturbations. Local recurrence was assessed clinically and by ultrasound through 12 months. Secondary endpoints were prespecified as descriptive/exploratory. For proportions (complications and 12‑month local recurrence), we report Wilson 95% confidence intervals and avoid formal hypothesis testing given the anticipated low event counts. Secondary outcomes were time-course changes in CEA and CA 125, technical success (complete real-time sonographic removal), 30-day complications graded by Clavien-Dindo, percentage lesion-volume reduction at one month, BREAST-Q satisfaction score at one and three months, and ultrasound-confirmed local recurrence at 12 months. Hematoma was defined as a post-operative collection > 30 mL on ultrasound, and cosmetic satisfaction ≥ 80 on the BREAST-Q (0–100) scale was categorized as “excellent.”

#### Confounding considerations

Paired design mitigates between-subject confounding; effect modification explored for menopausal status and lesion size (> 20 mm). Hormonal therapy/cycle phase and inflammatory markers were not collected; CA 125 treated as exploratory in premenopausal women.

### Laboratory methods

Venous blood was drawn from the antecubital vein, clotted 30 min, and centrifuged at 2,000 g for 10 min. Serum was analyzed within 2 h on a Cobas e802 electrochemiluminescence analyzer (Roche Diagnostics) with manufacturer CA 15 − 3, CEA, and CA 125 kits (analytic ranges: 0.5–200 U/mL; 0.2–100 ng/mL; 1–500 U/mL). Intra- and inter-assay CVs were < 5%. When immediate testing was not feasible, aliquots were stored at − 80 °C for ≤ 7 days. The laboratory performed two-level internal QC each run using standard acceptance rules; no additional validation beyond routine QC was undertaken.

#### Sample-size calculation

Using a paired-mean formula for non-inferiority with δ = 2 U/mL, standard deviation 6 U/mL from a pilot series (*n* = 30), α = 0.05 and 80% power, 71 participants were required; allowing 10% attrition, the target enrolment was set at 80.

### Data collection and management

Data were entered into REDCap v13 with role-based access; 10% of entries were source-verified by an independent monitor. Missing values ≤ 5% per variable were managed by listwise deletion; when > 5%, multiple imputation by predictive mean matching (m = 5) was used before analysis. All data were anonymized using coded identifiers and stored on an encrypted institutional server; the full de-identified data set and statistical code are available on request.

### Statistical analysis

Primary endpoint: within-patient change in CA 15 − 3 at 3 months. Noninferiority was met if the upper bound of the two-sided 95% CI for the mean paired difference (3 mo − baseline) was < + 2 U/mL (one-sided α = 0.025). Continuous data were presented as mean ± SD or median (IQR), categorical data were presented as number and percentage. For each biomarker, a Greenhouse–Geisser repeated-measures ANOVA tested overall time effects with prespecified baseline-vs-timepoint contrasts Holm-adjusted. Proportions use Wilson 95% CIs. Analyses were performed using R (Version 4.4).

The primary analysis used complete cases, with multiple imputation (m = 5; Rubin pooling) as sensitivity. Robustness was further assessed by a δ-adjusted tipping-point analysis (adding offsets to missing 3-month CA 15 − 3 until the upper 95% CI crossed + 2 U/mL), by TOST equivalence (± 2 U/mL), and by alternative NI margins (+ 1, + 3 U/mL).

Paired comparisons mitigate time-invariant confounding; CA 125 was interpreted cautiously (time-varying influences in premenopausal women). No routine multivariable adjustment was applied to secondary endpoints. We emphasize precision (Wilson CIs) and prespecified effect-modification checks (menopausal status; lesion size > 20 mm). Linear regression explored predictors of percentage lesion-volume reduction. Two-sided *p* < 0.05 unless adjusted.

## Results

### Participant flow and study cohort

**Fig. 1:**
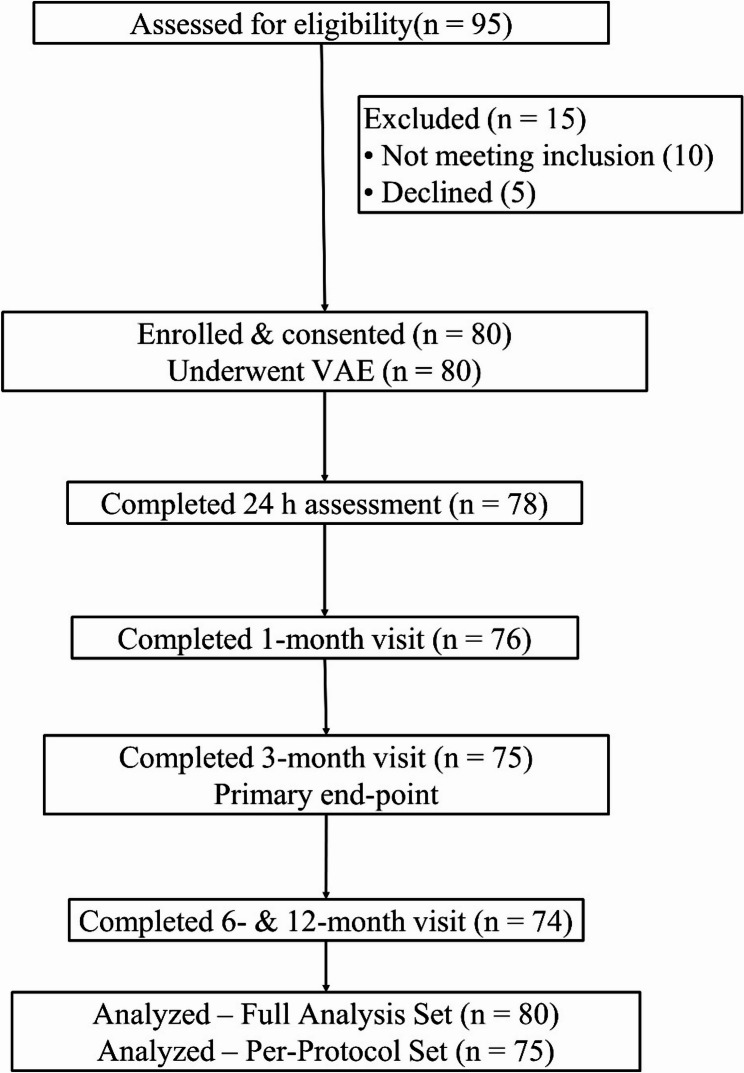
Patient flow diagram. Of 95 women screened for eligibility, 15 were excluded (10 did not meet inclusion criteria; 5 declined participation). The remaining 80 participants underwent ultrasound-guided Mammotome vacuum-assisted excision (VAE). Follow-up completion rates were 98% at 24 h (*n* = 78), 95% at 1 month (*n* = 76) and 94% at 3 months—the predefined primary end-point (Per-Protocol Set, *n* = 75). Seventy-four participants (93%) completed 6- and 12-month assessments. All 80 patients were included in the Full Analysis Set; 75 formed the Per-Protocol Set used for primary efficacy analyses

Of 95 women screened, 15 were excluded, leaving 80 who underwent ultrasound-guided Mammotome VAE and comprised the Full Analysis Set; 78 (98%) completed the 24-h assessment, 76 (95%) attended the 1-month visit, 75 (94%) reached the 3-month primary end-point and formed the Per-Protocol Set, and 74 (93%) completed 6- and 12-month follow-up (Fig. 1).

### Baseline demographic and clinical characteristics

The cohort’s mean age was 32 ± 8 years with a BMI of 22.6 ± 3.2 kg/m; most were pre-menopausal (85%) and multiparity was present in 38%. Median lesion count was 1 (IQR 1–2), largest-diameter 17 ± 6 mm, and BI-RADS categories were 3 in 62 patients and 4a in 18; histology confirmed fibroadenoma in 75%, adenosis in 19%, and benign phyllodes in 6%. Baseline tumor-marker means were 12.0 ± 4.0 U/mL for CA 15 − 3, 2.10 ± 1.10 ng/mL for CEA and 16 ± 6 U/mL for CA 125 (Table 1 ).Technical success was 98.8% (79/80) with a mean procedure time of 18 ± 5 min; minor 30‑day complications occurred in 7/80 (8.8%), mostly grade I, and 24‑h pain was low (VAS 2.3 ± 1.1) (Table 2).

**Table 1:** Baseline demographic and lesion characteristics

Variable	Value
Age (year), mean ± SD	32 ± 8
BMI (kg/m^2^), mean ± SD	22.6 ± 3.2
Pre-menopausal, n (%)	68 (85%)
Multiparity, n (%)	30 (38%)
Lesion count per patient, median (IQR)	1 (1–2)
Largest lesion diameter, mm	17 ± 6
BI-RADS category, n	
3	62
4a	18
Pathology subtype, n (%)	
Fibroadenoma	60 (75%)
Adenosis	15 (19%)
Benign phyllodes	5 (6%)
Baseline CA 15 − 3 (U/mL), mean ± SD	12 ± 4
Baseline CEA (ng/mL), mean ± SD	2.1 ± 1.1
Baseline CA 125 (U/mL), mean ± SD	16 ± 6

**Table 2:** Procedural details and early complications

Outcome	Statistic	95% CI
**Technical success**	79/80 (98.8%)	93.3%–99.9%
Procedure time, min	18 ± 5	
Excised tissue volume, cm³	2.4 ± 1.1	
Local anesthetic used, mL	14 ± 4	
**Complications ≤ 30 days**		
Bleeding > 30 mL	1 (1.3%)	0.2%–6.7%
Hematoma	3 (3.8%)	1.3%–10.5%
Infection	1 (1.3%)	0.2%–6.7%
Sensory change	2 (2.5%)	0.7%–8.7%
Clavien-Dindo grade I/II	6/1	
Pain VAS at 24 h	2.3 ± 1.1	

### Procedural metrics

**Fig. 2:**
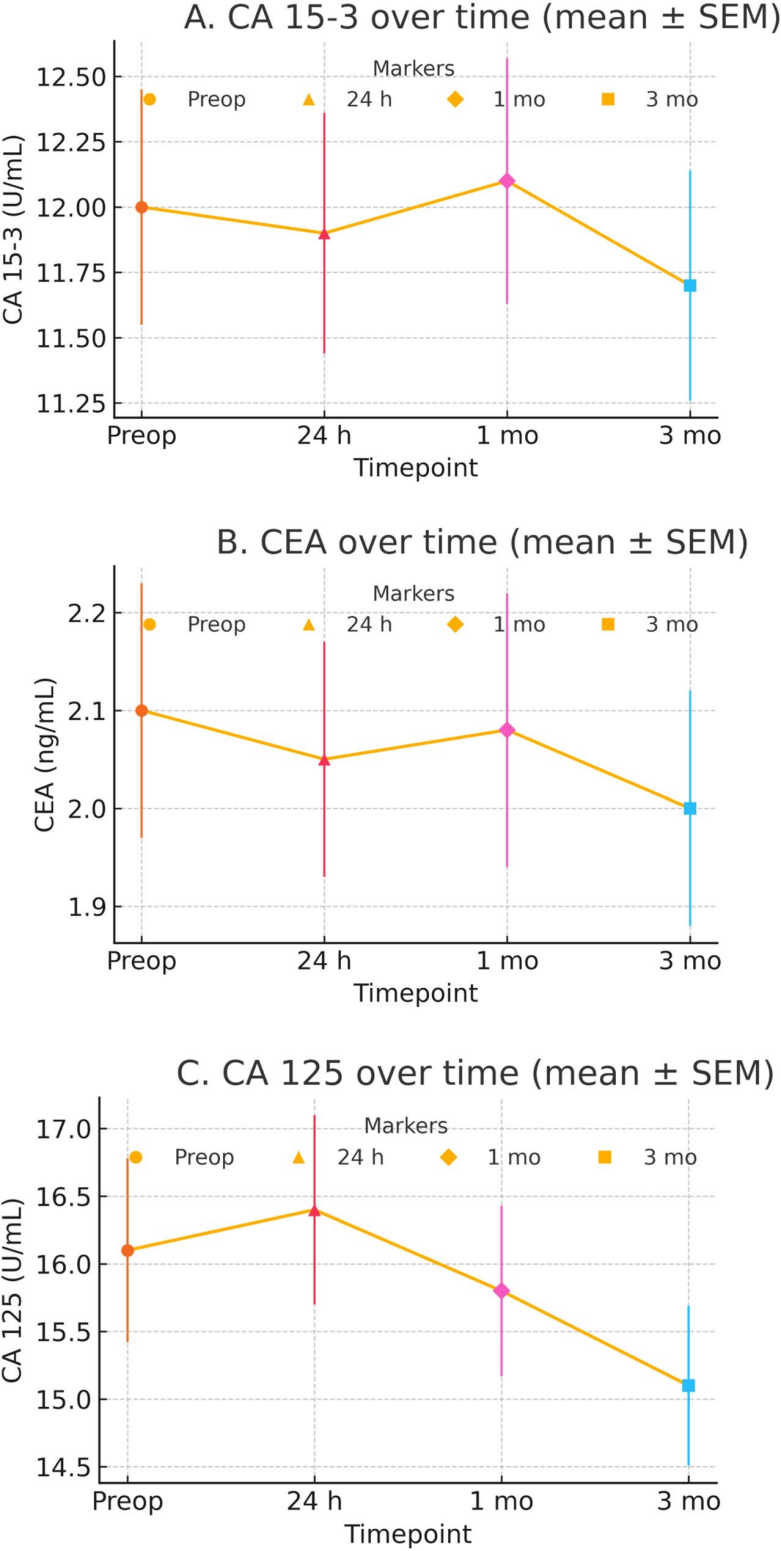
Peri‑operative trajectories of (**A**) CA 15‑3, (**B**) CEA, and (**C**) CA 125 (mean ± SEM). Repeated‑measures ANOVA (Greenhouse–Geisser): CA 15‑3, *p* = 0.30; CEA, *p* = 0.44. For CA 125, the baseline‑vs‑3 months contrast was nominally significant (*p* = 0.040) but not after multiplicity adjustment

Technical success, defined as complete real-time ultrasonographic removal, was achieved in 79/80 cases (98.8%, 95% CI 93.3–99.9%). Average operative time was 18 ± 5 min, excised tissue volume 2.4 ± 1.1 cm^3^ and local anesthetic consumption 14 ± 4 mL (Fig. 2).

Seven patients (8.8%, 95% CI: 4.31–17.0%) experienced Clavien-Dindo I–II events: one bleeding episode > 30 mL (1.3%, 95% CI: 0.23–6.74%), three hematomas (3.8%, 95% CI: 1.29–10.46%), one superficial infection (1.3%, 95% CI: 0.23–6.74%) and two transient nipple-areolar sensory changes (2.5%, 95% CI: 0.69–8.67%); mean 24-h pain score was 2.3 ± 1.1 on a 10-cm VAS, and no serious adverse events or conversions occurred (Fig. 2).

### Primary end-point – change in serum CA 15 − 3

Mean CA 15 − 3 showed no significant change (mean difference − 0.3 U/mL, 95% CI − 0.8 to 0.2; *p* = 0.24), and the confidence interval lay wholly within the prespecified ± 2 U/mL non-inferiority margin (Table 3). No patient exceeded the laboratory upper‑reference limit at any timepoint. Sensitivity analyses using alternative NI margins (+ 1 and + 3 U/mL) and a TOST check (± 2 U/mL) yielded concordant conclusions (Supplementary Table S1).

**Table 3:** Peri-operative serum tumor markers

Time-point	CA 15 − 3 (U/mL)	CEA (ng/mL)	CA 125 (U/mL)
Pre-operative (*n* = 80)	12.0 ± 0.45	2.10 ± 0.13	16.1 ± 0.68
24 h post-VAE (*n* = 78)	11.9 ± 0.46	2.05 ± 0.12	16.4 ± 0.70
1 month (*n* = 76)	12.1 ± 0.47	2.08 ± 0.14	15.8 ± 0.63
3 months – primary end-point (*n* = 75)	11.7 ± 0.44	2.00 ± 0.12	15.1 ± 0.59

No time-point mean exceeded the local upper-normal limit for any marker

Omnibus repeated‑measures ANOVA across time (Greenhouse–Geisser): CA 15‑3 *p* = 0.30; CEA *p* = 0.44; CA 125 *p* = 0.040 (not significant after Holm adjustment)

*SEM* standard error of the mean, *VAE* vacuum-assisted excision

### Secondary biomarker outcomes

No significant time effect for CA 15 − 3 (*p* = 0.30) or CEA (*p* = 0.44); CA 125 fell by 1.0 U/mL at 3 months (*p* = 0.040) but this was non-significant after Bonferroni correction (α_adj = 0.017); no patient exceeded the local upper-normal limit for any marker at any time point (Table 4). Predefined baseline‑vs‑timepoint contrasts were Holm‑adjusted within biomarker. No adjusted contrast was significant (Supplementary Table S2).

**Table 4:** Primary End-points

End-point	Baseline	3 months	Mean Δ (95% CI)	*p*-value^1^
CA 15 − 3, U/mL	12.0 ± 4.0	11.7 ± 3.8	–0.3 (–0.8 to 0.2)	0.244
CEA, ng/mL	2.10 ± 1.10	2.00 ± 1.02	–0.1 (–0.3 to 0.1)	0.312
CA 125, U/mL	16.1 ± 6.0	15.1 ± 5.9	–1.0 (–1.9 to − 0.1)	0.040^2^

Decision rule: Mean paired difference is shown with two‑sided 95% CI. Noninferiority for CA 15‑3 was prespecified if the upper 95% CI bound < + 2 U/mL (equivalent to one‑sided α = 0.025). Observed Δ = − 0.3 U/mL (–0.8 to + 0.2) meets this rule

^1^ Two‑sided paired t‑test; Wilcoxon signed‑rank test if non‑normal

^2^ Not significant after Bonferroni adjustment (α_adj = 0.017)

Stratified estimates of ΔCA 15‑3 by pathology, menopausal status, and lesion size were broadly consistent with the overall effect (Supplementary Table S3).

### Missing data and sensitivity analyses

Timepoint‑level biomarker completeness was high (baseline 100%; 24 h: 97.5%; 1 month: 95.0%; 3 months: 93.8%), corresponding to an overall biomarker missing data rate of ≈ 4% (Supplementary Table S4). Complete‑case and MI estimates were nearly identical for the primary endpoint and all biomarker time‑courses (Supplementary Table S5). In δ‑adjusted analyses, noninferiority remained robust to δ values of + 0.5, + 1.0, and + 2.0 U/mL applied to missing 3‑month CA 15‑3 values. Results were similar for CEA and CA 125 (Supplementary Table S5).

Local recurrence 1/74 (1.4%) had a Wilson 95% CI 0.24–7.27%; with *n* ≈ 190 a 2% event rate could be estimated to ± 2%, and with *n* ≈ 750 to ± 1% at 95% confidence. Complications 8.8% (7/80) yielded a Wilson 95% CI of roughly 3.8–17.1%. These intervals convey the available precision more appropriately than “observed power” (Supplementary Table S6).

### Cosmetic satisfaction and lesion-volume reduction

Mean BREAST-Q satisfaction score was 88 ± 8 at 1 month and 90 ± 7 at 3 months, with 85% of women rating their cosmetic outcome “excellent” (score ≥ 80); ultrasound demonstrated a 93% ± 6% median lesion-volume reduction one month post-procedure, and linear regression revealed no significant predictors of incomplete volume reduction (Table 5).

**Table 5:** Secondary End-points

End-point	3 months
BREAST-Q score (0–100)	90 ± 7
Lesion-volume reduction, % (1 mo)	93 ± 6
Local recurrence at 12 mo	1/74 (1.4%, 95% CI: 0.2–7.3%)^1^

Proportions are reported with Wilson 95% CIs

^1^ 95% CI was calculated by the Wilson method

## Discussion

This prospective cohort demonstrated great success with minor morbidity and favorable 12‑month local control, while serial measurements showed no clinically meaningful elevation of CA 15‑3, CEA, or CA 125 through 3 months. Crucially, serial measurements showed no clinically meaningful elevation of systemic tumor markers—CA 15 − 3, CEA or CA 125. Cosmetic satisfaction was uniformly high, and ultrasound documented a > 90% reduction in lesion volume one month after excision. Collectively, these data confirm that Mammotome VAE is highly effective at local tumor eradication has no measurable systemic impact on circulating tumor-associated antigens.

Our findings confirm and extend the robust body of evidence demonstrating that ultrasound-guided VAE consistently delivers technical success rates above 95% and low complication profiles for benign breast lesions. Our observation is similar with report for fibroadenoma and benign phyllodes tumor cohorts [5, 11, 12]. Patient-reported BREAST-Q satisfaction in our series also aligns with the satisfaction rate in large databases [6, 13] and surpasses that documented for open lumpectomy [12]. Consistent with the limited biomarker literature, our demonstration of stable CA 15 − 3 and CEA levels, coupled with a small, clinically irrelevant decline in CA 125, reproduces the pattern reported in smaller cohorts of fibroadenoma and adenosis [14–17]. Finally, the low local-recurrence rate at twelve months sits comfortably within the 0–4% range recorded in multicenter registries [18–20].

Our cohort’s baseline CA 15‑3 was within the laboratory reference range yet higher than the median values reported in some benign‑disease series [21]. Several factors likely contribute: (i) assay/platform differences and calibrators, which yield known method‑specific offsets; (ii) summary metric differences (means vs. medians/IQRs), and (iii) population mix—the present cohort was predominantly premenopausal with fibroadenoma/adenosis lesions. Crucially, our inference relies on the paired differences: the upper 95% CI for the mean difference at 3 months was + 0.20 U/mL, well below the + 2 U/mL noninferiority margin. This indicates stability irrespective of the starting absolute value.

The biological plausibility of these results rests on the negligible baseline expression of circulating tumor antigens in benign epithelial lesions such as fibroadenoma and intraductal papilloma [22, 23]. VAE removes tissue en bloc and seals the parenchymal cavity under negative pressure, minimizing cellular spillage and systemic release of antigens. Animal studies corroborate that limited parenchymal trauma markedly reduces hematogenous dissemination [24]. Our benign VAE cohort showed no measurable rise in circulating tumor‑associated antigens through 3 months. In contrast, studies of open breast surgery demonstrate short‑lived systemic inflammatory responses, with peri‑operative IL‑6 and CRP increases that correlate with operative injury and are attenuated by regional anesthesia or glucocorticoids [25, 26]. Similarly, in core‑needle biopsy of breast cancer, tissue‑level analyses reveal localized inflammatory cell recruitment and higher tumor‑cell proliferation adjacent to the biopsy tract [27]. These reports support the biological plausibility that tissue‑sparing, closed‑cavity excision could limit systemic effects. However, they derive from cancer populations and evaluate cytokines or histology, not serum tumor‑antigen kinetics, and therefore are presented here as mechanistic context rather than direct comparators.

Clinically, the convergence of high technical efficacy, excellent cosmesis and biomarker stability lends strong support to guideline recommendations that position VAE as first-line therapy for BI-RADS 3/4a fibroadenomas and similarly classified benign tumors ≤ 30 mm [29]. Our data also argue against routine postoperative testing of CA 15 − 3, CEA or CA 125 when baseline values are normal, a change that can curb costs and reduce patient anxiety without compromising safety [28]. This recommendation is conditional on normal preoperative markers and benign histology and should not supersede clinical judgment in patients with elevated or rising baseline values, new concerning symptoms, or higher‑risk pathology. Notably, contemporary oncology guidelines do not recommend serial CA 15‑3 for routine follow‑up even after treatment for early breast cancer, underscoring the limited role of these markers in low‑risk contexts [28, 29]. Our cohort did not collect direct cost or time‑to‑normal‑activity data, so we cannot claim cost‑effectiveness or faster recovery. However, we observed local anesthesia, a mean procedure time of 18 ± 5 min, low 24‑h pain (2.3 ± 1.1/10), and 8.8% Clavien–Dindo I–II events—findings that are consistent with efficient procedural care but should be interpreted strictly as descriptive proxies. A formal economic evaluation with prospectively captured costs and patient‑reported return‑to‑activity/work is needed to quantify any practice advantages over open excision.

This study was conducted at a single center with a single operator and without a concurrent comparator arm. As a result, although we demonstrate biomarker stability after VAE, we cannot exclude that similar stability might also follow other excisional techniques. Our nonrandomized design also permits residual selection bias, despite consecutive enrollment, predefined eligibility, objective laboratory endpoints, low rate of missing data with sensitivity checks, and high follow‑up. The cohort’s lesion spectrum further constrains generalizability to larger or higher‑risk lesions. The biomarker assessment was restricted to 3 months, which addresses peri‑operative systemic effects but does not establish longer‑term biological neutrality. Therefore, late events—particularly in benign phyllodes tumors—may not yet be captured. While the paired, within‑patient design reduces time‑invariant confounding, time‑varying factors were not fully addressed. We did not standardize or prospectively capture menstrual‑cycle phase or exogenous hormonal therapy, and we did not collect CRP or similar inflammatory markers; these omissions particularly affect interpretation of CA 125 in premenopausal women.

Our results support Mammotome VAE as a locally effective, minimally invasive option for appropriately selected benign lesions and show no measurable increases in CA 15‑3, CEA, or CA 125 through 3 months. Dedicated multicenter comparative evaluations are warranted.

## Supplementary Information

Supplementary Material 1.

## Data Availability

Data sets generated during the current study are available from the corresponding author on reasonable request.
