# Supplementary material for: Clinical outcomes of ultrasound-guided mammotome vacuum-assisted excision for benign breast tumors and its impact on serum tumor markers
Source: BMC Surg. 2025 Nov 21;25:565. doi: 10.1186/s12893-025-03293-x (PMC12639909; doi:10.1186/s12893-025-03293-x)
Supplement: Supplementary file 1 — Supplementary Material 1. [file 12893_2025_3293_MOESM1_ESM.docx]

**Supplementary Table S1. CA 15‑3 Noninferiority Sensitivity and TOST Equivalence**

| **Analysis** | **Decision rule** | **Margin/Bounds** | **how far the upper 95% CI is below the +margin** | **Conclusion** |
| --- | --- | --- | --- | --- |
| **Directional NI** | Upper 95% CI < +margin | +1 U/mL | 0.80 U/mL (1.00 − 0.20) | Noninferior |
| **Directional NI** | Upper 95% CI < +margin | +2 U/mL *(primary)* | 1.80 U/mL (2.00 − 0.20) | Noninferior |
| **Directional NI** | Upper 95% CI < +margin | +3 U/mL | 2.80 U/mL (3.00 − 0.20) | Noninferior |
| **TOST equivalence** | 90% CI entirely within ±2 U/mL | ±2 U/mL | — | Equivalent *(90% CI ≈ –0.72 to +0.12 U/mL)* |

**Supplementary Table S2. Baseline‑vs‑Timepoint Contrasts with Holm Adjustment**

|  | **Mean Δ (timepoint – baseline), (95% CI)** | **p (unadjusted)** | **p (Holm, within biomarker)** |
| --- | --- | --- | --- |
| **CA 15‑3 (U/mL)** |  |  |  |
| Baseline vs 24 h | –0.10 (–0.59 to +0.39) | 0.453 | 0.90 |
| Baseline vs 1 mo | +0.10 (–0.40 to +0.60) | 0.621 | 0.90 |
| Baseline vs 3 mo | –0.30 (–0.80 to +0.20) | 0.240 | 0.72 |
| **CEA (ng/mL)** |  |  |  |
| Baseline vs 24 h | –0.05 (–0.23 to +0.13) | 0.486 | 0.98 |
| Baseline vs 1 mo | –0.02 (–0.20 to +0.16) | 0.784 | 0.98 |
| Baseline vs 3 mo | –0.10 (–0.28 to +0.08) | 0.309 | 0.93 |
| **CA 125 (U/mL)** |  |  |  |
| Baseline vs 24 h | +0.30 (–0.50 to +1.10) | 0.657 | 0.68 |
| Baseline vs 1 mo | –0.30 (–1.10 to +0.50) | 0.342 | 0.68 |
| Baseline vs 3 mo | –1.00 (–1.90 to –0.10) | 0.040 | 0.12 |

**Supplementary Table S3. Exploratory Subgroup Estimates for ΔCA 15‑3**

| **Subgroup** | **n (paired)** | **Mean Δ CA 15‑3 (U/mL)** | **95% CI** |
| --- | --- | --- | --- |
| **Pathology** |  |  |  |
| **Fibroadenoma** | 56 | –0.30 | –0.88 to +0.28 |
| **Adenosis** | 14 | –0.20 | –1.36 to +0.96 |
| **Benign phyllodes** | 5 | –0.10 | –2.04 to +1.84 |
| **Menopausal status** |  |  |  |
| **Premenopausal** | 63 | –0.30 | –0.85 to +0.25 |
| **Postmenopausal** | 12 | –0.40 | –1.65 to +0.85 |
| **Largest lesion** |  |  |  |
| **> 20 mm** | 28 | –0.40 | –1.22 to +0.42 |
| **≤ 20 mm** | 47 | –0.20 | –0.83 to +0.43 |

**Supplementary Table S4.** **Missing data by biomarker and timepoint**

| **Biomarker** | **Baseline (n=80)** | **24 h (n=78)** | **1 mo (n=76)** | **3 mo (n=75)** | **Overall missing (%)** |
| --- | --- | --- | --- | --- | --- |
| **CA 15‑3** | 0/80 (0.0%) | 2/80 (2.5%) | 4/80 (5.0%) | 5/80 (6.3%) | ~4.0% |
| **CEA** | 0/80 (0.0%) | 2/80 (2.5%) | 4/80 (5.0%) | 5/80 (6.3%) | ~4.0% |
| **CA 125** | 0/80 (0.0%) | 2/80 (2.5%) | 4/80 (5.0%) | 5/80 (6.3%) | ~4.0% |

**Supplementary Table S5.** **Primary endpoint robustness: complete‑case vs MI (m = 5) and δ‑adjusted tipping‑point analysis (CA 15‑3)**

| **Analysis set** | **Mean Δ (U/mL)** | **95% CI** | **NI rule (upper 95% CI < +2 U/mL)** | **90% CI (TOST)** | **TOST (±2)** |
| --- | --- | --- | --- | --- | --- |
| **Complete‑case (n = 75)** | –0.30 | –0.80 to +0.20 | **Meets NI** | –0.72 to +0.12 | **Equivalent** |
| **MI m = 5 (pooled)** | –0.29 | –0.79 to +0.21 | **Meets NI** | –0.71 to +0.15 | **Equivalent** |
| **MI + δ = +0.5** | –0.26 | –0.76 to +0.24 | **Meets NI** | — | — |
| **MI + δ = +1.0** | –0.23 | –0.73 to +0.27 | **Meets NI** | — | — |
| **MI + δ = +2.0** | –0.17 | –0.67 to +0.33 | **Meets NI** | — | — |
| **Theoretical tipping‑point δ*** | — | — | **≈ +29 U/mL** | — | — |

* δ is the **additive offset** applied to **missing 3‑month** CA 15‑3 values only. Given missing data of **6.3%** at 3 months, δ ≈ **+29** U/mL would be required to push the **upper 95% CI** beyond **+2 U/mL**—far outside plausible analytic/biologic ranges.

**Supplementary Table S6.** **Precision (not post‑hoc power) for selected secondary outcomes**

| **Outcome** | **Observed estimate** | **95% CI** |
| --- | --- | --- |
| **Local recurrence (12 mo)** | 1/74 (1.4%) | 0.24–7.27% (Wilson) |
| **Any complication (30 d)** | 7/80 (8.8%) | ~3.8–17.1% (Wilson) |
| **Δ CA 125 (3 mo)** | –1.0 U/mL | –1.9 to –0.1 |
